# A Combined Strategy to Improve the Development of a Coral Antivenom Against *Micrurus* spp.

**DOI:** 10.3389/fimmu.2019.02422

**Published:** 2019-10-21

**Authors:** Karen Larissa Pereira de Castro, Letícia Lopes-de-Souza, Daysiane de Oliveira, Ricardo Andrez Machado-de-Ávila, Ana Luiza Bittencourt Paiva, Cláudio F. de Freitas, Paulo Lee Ho, Carlos Chávez-Olórtegui, Clara Guerra-Duarte

**Affiliations:** ^1^Department of Biochemistry and Molecular Biology, University of Texas Medical Branch, Galveston, TX, United States; ^2^Departamento de Bioquímica, Instituto de Ciências Biológicas, Universidade Federal de Minas Gerais, Belo Horizonte, Brazil; ^3^Universidade de Extremo Sul Catarinense, Criciúma, Brazil; ^4^Fundação Ezequiel Dias, Belo Horizonte, Brazil; ^5^Instituto Butantan, São Paulo, Brazil

**Keywords:** antivenom, synthetic peptides, *Micrurus*, snake, epitopes, three-finger toxins, phospholipase A_2_

## Abstract

Accidents involving *Micrurus* snakes are not the most common ones but are noteworthy due to their severity. Victims envenomed by *Micrurus* snakes are at high risk of death and therefore must be treated with coral antivenom. In Brazil, the immunization mixture used to fabricate coral antivenom contains *Micrurus frontalis* and *Micrurus corallinus* venoms, which are difficult to be obtained in adequate amounts. Different approaches to solve the venom limitation problem have been attempted, including the use of synthetic and recombinant antigens as substitutes. The present work proposes a combined immunization protocol, using priming doses of *M. frontalis* venom and booster doses of synthetic B-cell epitopes derived from *M. corallinus* toxins (four three-finger toxins-3FTX; and one phospholipase A_2_-PLA_2_) to obtain coral antivenom in a rabbit model. Immunized animals elicited a humoral response against both *M. frontalis* and *M. corallinus* venoms, as detected by sera reactivity in ELISA and Western Blot. Relevant cross-reactivity of the obtained sera with other *Micrurus* species (*Micrurus altirostris, Micrurus lemniscatus, Micrurus spixii, Micrurus surinamensis)* venoms was also observed. The elicited antibodies were able to neutralize PLA_2_ activity of both *M. frontalis* and *M. corallinus* venoms. *In vivo*, immunized rabbit sera completely protected mice from a challenge with 1.5 median lethal dose (LD_50_) of *M. corallinus* venom and 50% of mice challenged with 1.5 LD_50_ of *M. frontalis* venom. These results show that this combined protocol may be a suitable alternative to reduce the amount of venom used in coral antivenom production in Brazil.

## Introduction

Snakebite is a worldwide health problem, considered by the World Health Organization (WHO) as a neglected tropical disease (1). Almost 3 million snake envenomings, with 81,000–138,000 deaths, are officially reported per year. However, since most accidents occur in poor rural areas often devoid of medical care and proper data registry, this number is thought to be largely underestimated (2).

In Brazil, four genera are responsible for the medically relevant accidents: *Bothrops, Crotalus, Lachesis*, and *Micrurus* (3). Among them, elapid envenomation caused by snakes from the genus *Micrurus* are not the most common ones, but are noteworthy due to their severity, as more than 26% of the cases are considered to be severe (in bothropic accidents, the most prevalent ones, severe accidents correspond to only 7% of the cases) (4).

In human accidents caused by *Micrurus* snakes, there is substantial risk of neuromuscular blockage, with paralysis and respiratory failure leading to death. Even patients admitted with mild symptoms or even completely asymptomatic can progress to paralysis in a short time interval (5). Therefore, the treatment protocol recommended by the Brazilian Ministry of Health states that all victims of elapid accidents must receive 10 ampoules of coral antivenom, regardless of the severity of the initial symptoms presented (6).

Brazilian coral antivenom is produced from horse hyperimmunization with venom from the two species responsible for most accidents (7): *Micrurus frontalis* and *Micrurus corallinus*; but at least 33 other species are described in the country (8). Venom availability is an important bottleneck for antivenom production, since *Micrurus* snakes are relatively small, with reduced venom glands and lower venom yields compared to other snakes. While *Bothrops* snakes give around 80 mg of venom per milking, *Micrurus* venom yield is considerably lower. The amount of venom that can be extracted from a *Micrurus* snake can vary greatly depending on the species. It ranges from 3 mg for *M. corallinus* to 54 mg per milking in *M. suranimensis*, but venom yield average rarely exceeds 20 mg (7–10). Also, *M. corallinus* is a species particularly sensitive to captivity, with important dietary restrictions and disease susceptibility. Moreover, the acquisition of new snake specimens by antivenom producers animal husbandry has decreased over time, since it has been more difficult to find them in nature due to their fossorial habits and reduction of their natural habitat (9, 11).

Research efforts have been made to overcome these problems in coral antivenom production. Better animal management (11), strategies to enhance collected venom yields (9) and even a suggestion of using cross-neutralizing antivenom obtained from other species of Elapidae snakes (12) were proposed.

Another approach to address this problem is the use of synthetic substitutes to *M. corallinus* venom. In 2009, Leão and collaborators indicated some candidate molecules from its venom gland transcriptome analysis to represent *M. corallinus* venom in antivenom production. The toxin selection was based on abundance and representative variability. Three-finger toxins (3FTX) and phospholipases A2 (PLA2) accounted for more than 85% of the toxins expressed. Thus, cDNAs corresponding to four diverse 3FTXs and one PLA2 were applied in a preliminary immunization protocol. The selected antigens could induce specific antibodies, although venom recognition by the generated antibodies in ELISA was low (13).

Using the same five toxins from *M. corallinus* venom selected by Leão et al. (13), Castro et al. (14) performed epitope mapping of these antigens by SPOT technique and bioinformatic analysis. The combination of the mapping approaches of these five antigens resulted in the selection of nine sequences corresponding to putative epitopes, which were chemically synthesized. A mixture of these synthetic peptides was used to immunize rabbits. Anti-peptides antibodies were capable of neutralizing phospholipase A2 and lethal activities of *M. corallinus* venom, validating the potential application of these synthetic molecules in antivenom production. Ramos et al. (15) also used the antigens defined by Leão et al. (13) to map epitopes and proposed a genetic immunization protocol using DNA-strings and a multiepitopic protein. Serum derived from the genetic immunization protected mice challenged with *M. corallinus* venom.

Considering all previous efforts described above, the present work proposes a combined immunization protocol to produce a bivalent coral antivenom, using crude *M. frontalis* venom and substituting *M. corallinus* venom for the synthetic peptides validated by Castro et al. (14).

## Materials and Methods

### Animals and Venoms

*Micrurus* sp. venoms were kindly provided by Ezequiel Dias Foundation (FUNED): *M. frontalis, M. corallinus, M. lemniscatus, M. altirostris* and by Instituto National de Salud (Peru): *M. spixii, M. surinamensis*. Snakes' subspecies of the obtained venom samples were not specified by the donors. Lyophilized venoms were stored at −20°C in the dark. Prior to use, venoms were dissolved in ultra-pure water and protein content was determined by Lowry method (16), using bovine serum albumin as standard.

Female Swiss mice (18–22 g) and New Zealand female rabbits (2 kg) were maintained in Centro de Bioterismo of Instituto de Ciências Biológicas of Universidade Federal de Minas Gerais (UFMG), Brazil. All animals received food and water *ad libitum* under controlled environmental conditions.

This study was carried out in accordance with the principles of the Basel Declaration and recommendations of the Brazilian Council for the Control of Animal Experimentation (CONCEA). The protocol was approved by the Ethics Committee in Animal Experimentation from the Federal University of Minas Gerais (protocol 375/2012-CETEA/UFMG).

### Synthesis of Soluble Peptides

Epitope sequences mapped in the work of Castro et al. (14) (39PDDFTCVKKWEGGGRRV55, from 3FTX Mcor0100c, named Pep100; 37TCPAGQKICFKKWKKG52 and 64PKPKKDETIQCCTKNN79, from 3FTX Mcor0039c, named Pep039a and Pep039b, respectively; 22LECKICNFKTCPTDELRH39 and 54THRGLRIDRGCAATCPTVK72 from 3FTX Mcor0604c, named Pep604a and Pep604b; 28RHASDSQTTTCLSGICYKK45 and 58GCPQSSRGVKVDCCMRDK75, from Mcor0599c, named Pep599a and Pep599b, respectively and peptides 28NLINFQRMIQCTTRRSAW45 and 119NCDRTAALCFGRAPYNKNN137, from McorPLA2, named PepPLA2a and Pep- PLA2b) were synthesized by the Fmoc chemistry method on an automatic Multipep robot (Intavis). All internal cysteine residues were replaced by serines and a tyrosine was added to the N-terminus of the sequences which did not possess aromatic residues (Pep039b, Pep604a and b, and Pep599b) in order to allow quantification of peptides by absorbance at 280 nm. During the synthesis, peptides were immobilized on Rink Amide resin (Novabiochem). At the end of the synthesis, peptides were released from the resin, and side chain deprotection was carried out by trifluoroacetic acid treatment (95% TFA, 2.5% triisopropylsilane, and 2.5% water). All peptides were N-terminally acetylated and C-terminally amidated. After synthesis, peptides were lyophilized, and their purity was assessed by mass spectrometry in system MALDI-TOF/TOF (Autoflex III e Bruker Daltonics Inc.). The experimentally measured peptide masses differed from the theoretically expected by 1.012 Da ± 0.658 on average, indicating good synthesis quality.

### Immunization Protocols

**Figure 1:**
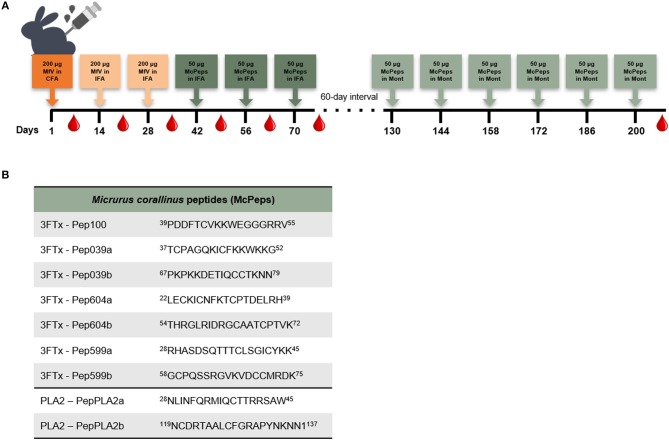
Immunization protocol. **(A)** Rabbits immunization scheme for coral antivenom alternative production. Doses were given at 2-week intervals with the immunogen described in the boxes above the line. Bleedings were performed 1 week after each dose, as signaled. **(B)** Peptides sequences derived from epitope prediction of *Micrurus corallinus* toxins, made by Castro et al. (14) used in the immunization protocol.

Adult New Zealand female rabbits were immunized as a proof-of-concept of the proposed immunization protocol for coral antivenom production (Figure 1A). After collection of non-immune sera, animals received an initial subcutaneous injection of 200 μg of *M. frontalis* crude venom in complete Freund's adjuvant (day 1). Two booster injections were made subcutaneously at intervals of 2 weeks with the same dose (200 μg) of *M. frontalis* venom in incomplete Freund's adjuvant. Two weeks after that, rabbits received three subcutaneous injections of 450 μg of the mixture of all synthetic peptides (Figure 1B) (50 μg of each peptide) in incomplete Freund's adjuvant, also at intervals of 2 weeks. Blood samples were drawn 1 week after each injection. After a break of 60 days, rabbits received six additional doses of 450 μg of the mixture of all peptides in Montanide adjuvant at intervals of 2 weeks. Blood samples were drawn 1 week after the last injection.

### IgG Purification

IgGs were purified from immunized rabbit's sera. Serum IgGs were concentrated by precipitation with ammonium sulfate and purified by affinity chromatography using a Protein A-Sepharose column (GE Healthcare), according to the protocol described by the GE Healthcare Bio-Sciences AB.

### Indirect ELISA Assays

Microtitration plates (Costar, USA) were coated overnight with either 0.5 μg/well of *Micrurus* venoms at 4°C or 1.0 μg/well of glutaraldehyde polymerized peptides at 37°C in carbonate buffer pH 9.6. After blocking (3% skimmed milk in PBS) and washing (0.05% Tween-saline), non-immune rabbit sera or immune sera were added in different dilutions and incubated for 1 h at 37°C. Plates were washed and incubated with anti-rabbit IgG conjugated with peroxidase (Sigma, USA) diluted 1:10,000, for 1 h at 37°C. After washing, OPD Peroxidase substrate (SIGMAFAST from Sigma-Aldrich) was added to the wells. The reaction was interrupted after 30 min using 20 μl of a 1:20 sulfuric acid solution. Absorbance values were determined at 490 nm using an ELISA plate reader (BIO-RAD, iMark models, EUA). Values represent the mean of two independent experiments.

### Western Blotting

*Micrurus* venoms were diluted in sample buffer under reducing conditions and SDS-PAGE was performed on 18% polyacrylamide gels. Protein bands were visualized by silver staining or transferred to nitrocellulose membranes for immunoblotting.

For western blot, gels were wet-transferred to nitrocellulose membranes overnight. The membrane was blocked with PBS-Tween 0.3% for 1 h. After washing three times for 5 min with PBS-Tween 0.05%, the membrane was incubated with either rabbit non-immune serum, immunized rabbit sera or commercial coral antivenom produced by FUNED, diluted 1:2,000 for 1 h. The membrane was washed (PBS-Tween 0.05%) three times and immunoreactive proteins were detected using anti-rabbit or anti-horse IgGs conjugated to peroxidase for 1 h at 37°C. After additional washes, reaction was detected using DAB/chloronaphthol substrate, according to the manufacturer's instructions.

### Phospholipase A2 Activity Determination

To analyze PLA2 activity, EnzChek® PLA2 Assay Kit (Life Technologies) was used. The experiment was made following EnzCheck's protocol, using 2 μg of either *M. frontalis* or *M. corallinus* venom. A solution of PLA2 10 U/mL in 1 × PLA2 reaction buffer was used as positive control and the same buffer without PLA2 was used as negative control. All assays were performed in duplicates. Means of the results from two independent experiments were calculated and plotted as percentage of activity, relative to the positive control.

### Neutralization Assays

#### Neutralization of Phospholipase Activity

PLA2 activity was determined using an indirect hemolytic assay described by Gutiérrez et al. (17). Samples with increasing concentrations of either *M. frontalis* or *M. corallinus* venom were prepared in a final volume of 15 microliters in PBS and added to 3 mm wells in agarose gels (0.8% in phosphate buffered saline, pH 8.1) containing 1.2% rabbit erythrocytes, 1.2% egg yolk as a lecithin source and 100 mM of CaCl2. After incubation at 37°C for 18 h in a wet chamber, hemolytic halos were measured. Then, the minimum phospholipase dose (MPD: the minimum concentration of venom which produced a hemolytic halo of 1 cm of diameter) was determined.

For assessing the PLA2 neutralizing potential of rabbits' IgG's, increasing concentrations of IgGs were pre-incubated with 1 MPD of either *M. frontalis* or *M. corallinus* crude venom at 37°C for 1 h and added to the 3 mm wells in agarose. The assay proceeded as described above. As controls, non-immune IgGs were incubated with venoms (C+) and a pool of IgG was incubated with PBS (C–).

#### Neutralization of Lethal Activity

For *in vivo* neutralization assays, 3 groups of 4 mice were used for each venom. Animals were injected intraperitoneally with 500 μl of a solution containing a dose corresponding to 1.5 LD50 of either *M. frontalis* (33 μg/20 g mouse) or *M. corallinus* (10.5 μg/20 g mouse) venom, pre-determined by Tanaka et al. (18) in PBS-BSA 0.1%, pre-incubated for 1 h at 37°C with either with 100 μl of a pool of sera from immunized rabbits, 100 μl coral antivenom or 100 μl of PBS. Dead animals were counted 48 h after the challenge.

## Results

### Immunocharacterization of Elicited Antibodies

To overcome difficulties in producing bivalent coral antivenom, we propose a combined protocol, using crude *M. frontalis* venom and synthetic peptides derived from *M. corallinus* toxins sequences. Two rabbits were immunized using the immunogen combination, as a proof-of-concept. The produced sera will be further named as anti-Ven_fro_/Pep_cor_, referring that the elicited antibodies are directed against crude venom of *M. frontalis* and peptides derived from toxin's sequences of *M. corallinus*.

**Figure 2:**
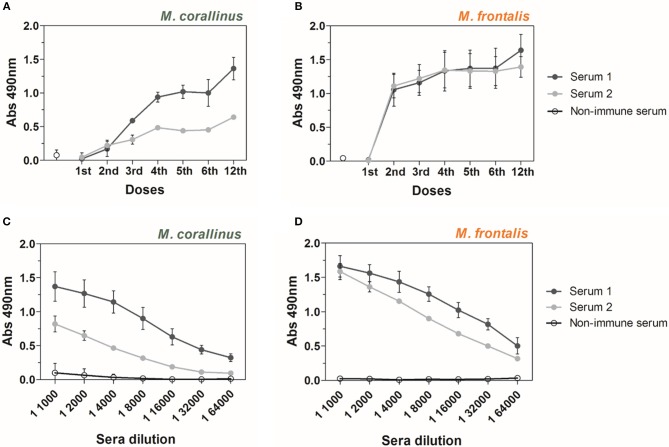
ELISA reactivity of anti-Ven_fro_/Pep_cor_ sera against *M. corallinus* and *M. frontalis* venoms. Graphs show ELISA reactivity of rabbit 1 and 2 anti-Ven_fro_/Pep_cor_ sera (Serum 1 and serum 2) against **(A)** *M. corallinus* venom and **(B)** *M. frontalis* venom. Plates were coated with 0.5 μg/well of each venom and incubated with sera of different immunization doses. Sera obtained after 12th dose were titrated (1:1,000–1:64,000) against **(C)** *M. corallinus* venom and **(D)** *M. frontalis* venom. Anti-horse peroxidase (1:10,000) was used as secondary antibody and Sigma OPD tablets detected recognition, according to manufacturer's instructions. Non-immune serum was used as negative control. Values are means ± SD of two independent experiments.

Antibody reactivity of anti-Ven_fro_/Pep_cor_ sera was assayed with both *Micrurus* venoms by ELISA, after each immunization dose given to animals (Figures 2A,B). Results show that the proposed immunization protocol induced antibodies able to recognize *M. frontalis* and *M. corallinus* venoms in both rabbits, although serum from rabbit 2 showed weaker binding to *M. corallinus* venom (Figure 2A). The first three doses in the protocol used crude *M. frontalis* venom. Therefore, the immune response against this venom increased more rapidly (after the 2nd dose) than against *M. corallinus*. On the other hand, antibody reactivity against *M. corallinus* continued to increase throughout the immunization protocol, whereas reactivity against *M. frontalis* plateaued after the 4th dose, with a slight increase after the 12th dose for serum 1. It is relevant to mention that *M. corallinus* peptides booster doses were able to keep serum reactivity against *M. frontalis*, even in the absence of this venom as antigen in the subsequent doses.

After the 12th dose, anti-Ven_fro_/Pep_cor_ sera were titrated against both venoms (Figures 2C,D). Antibody titers against *M. corallinus* were lower than against *M. frontalis*. Sera from rabbit 1 and 2 showed different reactivities against *M. corallinus* and were more homogenous against *M. frontalis*. However, both animals produced a satisfactory response against both venoms.

**Figure 3:**
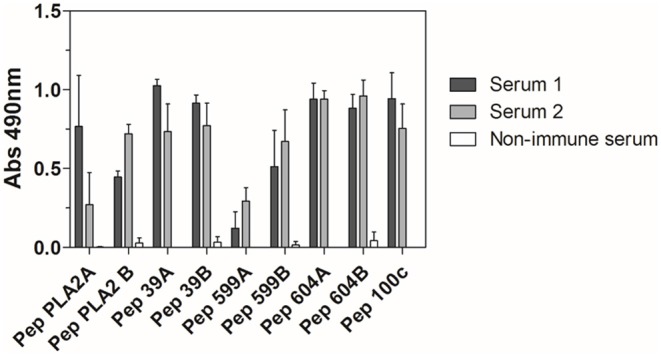
ELISA reactivity of anti-Ven_fro_/Pep_cor_ sera against *M. corallinus* peptides. Rabbit sera after 12th dose (diluted 1:100) were tested against individual peptides coated to ELISA plates (10 μg/well). Anti-horse peroxidase (1:10,000) was used as secondary antibody and Sigma OPD tablets detected recognition, according to manufacturer's instructions. Non-immune serum was used as negative control. Values are means ± SD of two independent experiments.

To further characterize the antigenicity of the peptides used in the immunization protocol, anti-Ven_fro_/Pep_cor_ sera reactivity against each individual peptide was also assessed. Except for Pep599a, all peptides were well recognized by both sera. Serum from rabbit 1 and rabbit 2 also presented similar reactivity against all peptides, with a marked difference detected only for PepPLA2a (Figure 3).

### Cross-Reactivity of Produced Sera With Other *Micrurus* Venoms

Along with *M. corallinus* and *M. frontalis*, other species from *Micrurus* genus also occur in Brazil. Since the precise identification of the exact species of the offender snake is rarely made, all elapid accidents are treated with the same antivenom. Therefore, it is relevant to test cross-reactivity of the produced anti-Ven_fro_/Pep_cor_ sera against other *Micrurus* venoms.

**Figure 4:**
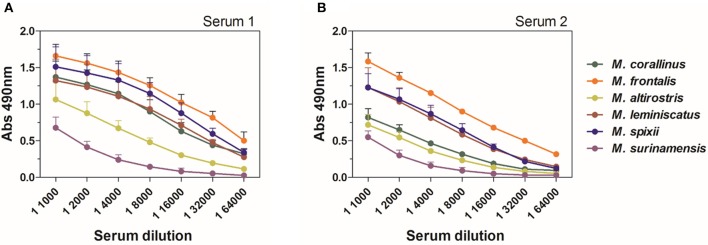
ELISA cross-reactivity of anti-Ven_fro_/Pep_cor_ sera against *Micrurus* venoms. **(A)** Shows results for reactivity of serum from rabbit 1 and **(B)** shows results for rabbit 2. Plates were coated with 0.5 μg/well of each *Micrurus* venom and incubated with different dilutions of rabbit sera after 12th dose (1:1,000–1:64,000). Anti-horse peroxidase (1:10,000) was used as secondary antibody and Sigma OPD tablets detected recognition, according to manufacturer's instructions. Non-immune serum was used as negative control. Values are means ± SD of two independent experiments.

In an ELISA assay, *M. lemniscatus* and *M. spixii* showed considerable cross-reactivity, being more strongly or equally recognized by anti-Ven_fro_/Pep_cor_ sera of both rabbits than *M. corallinus*. *M. altirostris* and *M. surinamensis*, showed a weaker, yet remarkable reactivity with both sera (Figure 4).

**Figure 5:**
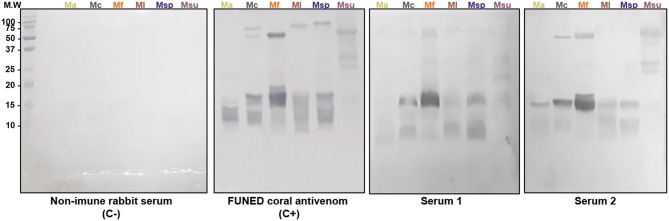
Western blot analysis of anti-Ven_fro_/Pep_cor_ sera against *Micrurus*. Venoms (20 μg) from *M. altirostris* (MaV), *M. corallinus* (McV), *M. frontalis* (MfV), *M. leminiscatus* (MlV), *M. spixii* (MspV), and *M. surinamensis* (MsuV) were submitted to SDS-PAGE 18% under reducing conditions. Molecular size markers (MWM) are indicated in the left (in kDa). Rabbit sera, including non-immune serum, and FUNED antivenom were diluted 1/2.000.

A Western Blot assay was also performed to characterize the immunoreactivity of anti-Ven_fro_/Pep_cor_ sera against several *Micrurus* venoms and compare them with the approved therapeutical bivalent coral antivenom produced by FUNED (Figure 5). As verified in the ELISA assays, serum from rabbit 1 showed a slightly more intense reaction with the venoms. FUNED coral antivenom reacted with bands above 38 KDa more intensely than the produced anti-Ven_fro_/Pep_cor_ rabbit sera.

**Figure 6:**
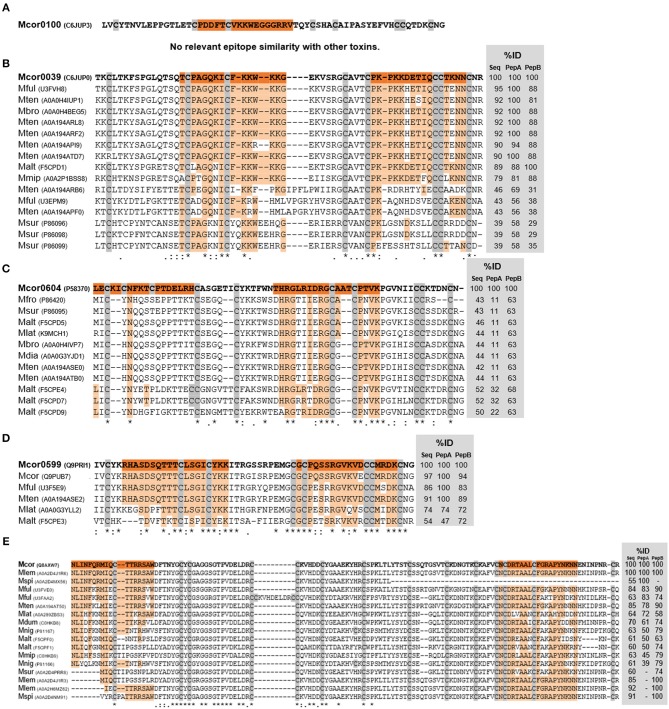
Alignments of *M. corallinus* toxins mature sequences. Alignment was performed with MUSCLE–EMBL. Conserved cysteines are highlighted in gray. Percentage of identity (%ID) with reference toxins was calculated using the tool EMBOSS Stretcher for pairwise sequence alignment using either their mature sequence or the specific sequences of the two selected peptides. The reference sequence from *M. corallinus* is in bold and mapped epitopes sequences are highlighted in orange. Aligned sequences are identified by their species initials and UNIPROT number in parenthesis. At the bottom alignment line, an (*) indicates positions which have a single, fully conserved residue; (:) indicates conservation between groups of strongly similar properties; (.) indicates conservation between groups of weakly similar properties. 3FTx toxins alignments are shown in **(A–D)** and PLA2 alignment is shown in **(E)**.

**Figure 7:**
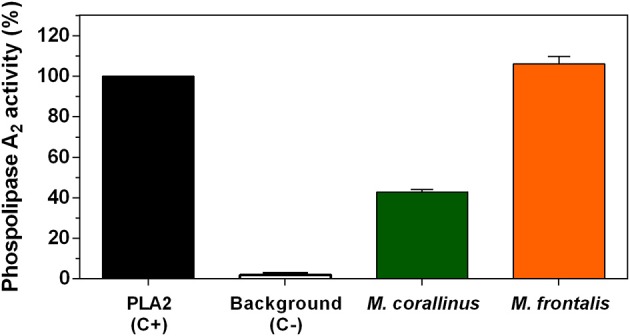
Phospholipase A2 activity. The EnzChek Phospholipase A2 Assay Kit was used to test *M. corallinus* and *M. frontalis* venoms (2 μg) according to the manufacturer's instructions. Fluorescence was determined at 490 nm excitation and 570 nm emission. Data are represented as the percentage of activity in relation to positive control (purified PLA2 from *Apis mellifera*). Values represent means ± SD of three independent experiments.

Toxins and mapped epitope sequences were aligned to other *Micrurus* 3FTx (Figures 6A–D) and PLA2 (Figure 6E) toxins available at UniProt to verify sequence similarities that could explain the sera cross-reactivities verified with the tested venoms. The alignments showed that not only toxins from the tested venoms but also from other *Micrurus* species have similar regions, ranging from 100 to 50% of identity, that can potentially explain cross-reaction of these venoms with anti-Ven_fro_/Pep_cor_ sera (Figures 6, 7). Pep100 and Pep604a did not show significant similarity to other *Micrurus* toxins and seems to be exclusive to *M. corallinus* venom.

### Neutralization Assays

Reactivity of the produced anti-Ven_fro_/Pep_cor_ sera toward different *Micrurus* venoms was well established using different techniques. However, antibody *in vitro* reactivity not always means efficient neutralization of toxic venom activities. To this end, neutralization assays of PLA2 activity and lethality were performed for *M. frontalis* and *M. corallinus* venoms.

**Figure 8:**
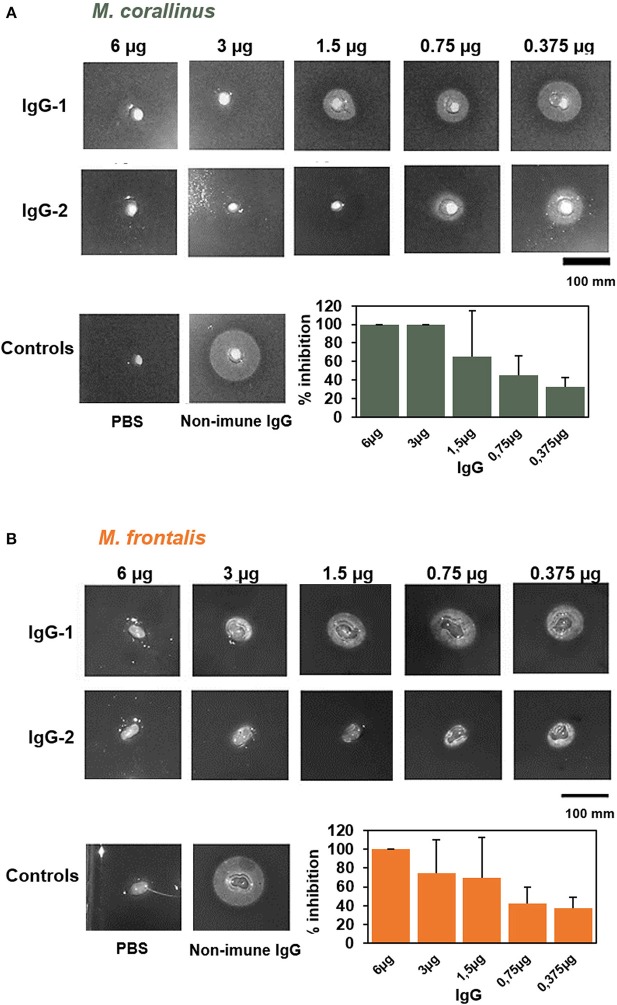
Neutralization of PLA2 activity by anti-Venfro/Pepcor purified IgGs. Neutralization of phospholipase activity of **(A)** *M. frontalis* or **(B)** *M. corallinus* venom by IgGs anti-Ven_fro_/Pep_cor_. An indirect hemolytic assay was performed, using 1 Minimum Phospholisic Dose (MPD) of each venom incubated with different amounts of IgG (6–0.375 μg). Non-immune IgG (6 μg) incubated with venom (1 MPD) and IgG incubated with PBS instead of venom were used as controls. The percentage of PLA2 activity inhibition was measured and results were plotted in a bar graph. Values are means ± SEM of two independent assays using the two different rabbit's IgGs.

First, PLA2 basal activity of both venoms was determined by a fluorometric (Figure 7) and by indirect hemolytic assays (Figure 8). *M. frontalis* showed stronger PLA2 activity in both methodologies. Two micrograms of *M. frontalis* venom matched positive control activity of 100%, even exceeding it a little, whereas *M. corallinus* barely achieved 40% of the C+ activity in the fluorometric assay.

The PLA2 neutralizing potential of the produced antibodies was tested in the indirect hemolytic assay, using different amounts of IgGs purified from anti-Ven_fro_/Pep_cor_ sera (6–0.375 μg) (Figure 8) incubated with 1 minimum phospholipase dose (MPD). This dose was established previously (14) for each venom: *M. frontalis* was 0.022 μg of venom and 1.84 μg for *M. corallinus* (almost 85-times less active than *M. frontalis*). We used purified IgGs instead of serum to avoid interference of other serum components in the assay. Phosphate buffer incubated with 6 μg of anti-Ven_fro_/Pep_cor_ IgGs was used as negative control and 1 MPD of each venom incubated with 6 μg of non-immune IgG was used as positive control. Consistent with its higher phospholipase activity, *M. frontalis* venom was less efficiently neutralized by anti-Ven_fro_/Pep_cor_ IgGs, achieving 100% inhibition only with the higher concentration of IgG tested. Contrary to what was observed in the immunocharacterization assays, IgGs from rabbit 2 seems to have a higher PLA2 neutralization potential than those from rabbit 1, confirming that antibody recognition of a given antigen does not mean necessarily antibody neutralization.

For the *in vivo* lethality neutralization (Table 1), we used 100 μl of a pool of both rabbits' sera incubated for 1 h at 37°C with an amount of venom equivalent to 1.5 LD50 of each *Micrurus* venom. Bivalent coral antivenom from FUNED was used as a positive control and PBS as a negative control. As expected, FUNED's antivenom fully protected animals from the venom challenge with both venoms, and the dose of 1.5 LD50 of both venoms incubated only with PBS killed 100% of injected mice. Anti-Ven_fro_/Pep_cor_ sera showed to have lethality neutralization potential and fully protected animals from the challenge with *M. corallinus* venom. However, *M. frontalis* venom was less efficiently neutralized by anti-Ven_fro_/Pep_cor_ sera, that protected only 50% of challenged animals. The assay was initially made with four animals. To confirm the difference of protection between *M. corallinus* and *M. frontalis* venom, the assay was repeated only for anti-Ven_fro_/Pep_cor_ sera and results were the same, indicating that indeed protection against *M. corallinus* venom lethality was higher when compared to *M. frontalis* venom.

**Table 1:** *In vivo* protection of anti-Ven_fro_/Pep_cor_ sera.

**Group**	**Survival/injected**	**% Survival**
***M. corallinus*** **(1.5 LD**_**50**_**)**		
Anti-Ven_fro_/Pep_cor_	8/8	100%
Coral antivenom (C+)	4/4	100%
PBS (C–)	0/4	0%
***M. frontalis*** **(1.5 LD**_**50**_**)**		
Anti-Ven_fro_/Pep_cor_	4/8	50%
Coral antivenom (C+)	4/4	100%
PBS (C–)	0/4	0%

*Amounts of M. corallinus or M. frontalis venom, corresponding to 1.5 DL50 were pre-incubated for 1 h at 37°C with 100 μl of either PBS (C–), commercial coral antivenom from FUNED (C+) or anti-Ven_fro_/Pep_cor_ sera. Animals were injected intraperitoneally, and deaths were recorded 48 h after injections*.

## Discussion

Bites caused by snakes from the genus *Micrurus* represent <1% of snakebite cases notified in Brazil, but most of the accidents are considered severe and antivenom administration is recommended primarily in all cases (19). Antivenom shortage is a worldwide health problem (20) but Brazil stands as an exception, being self-sufficient in antivenom production (21–23). Nonetheless, the production of Brazilian coral antivenom specifically faces drawbacks and its production and quality control is limited, due to the difficulty in obtaining enough amounts of coral venom (11, 24, 25).

To illustrate in figures the venom shortage in coral antivenom production, in the year of 2019, Ezequiel Dias Foundation (FUNED) has obtained until August a total of 133 mg of venom from 11 specimens of *M. frontalis* kept in the Foundation's Serpentarium. However, to perform the immunization protocol for coral antivenom production to supply the yearly national demand, it is necessary at least 450 mg of venom, considering the quality control tests. These numbers show that the amount of venom that has been obtained so far represents <30% of the amount needed for the production of coral antivenom. Venom yield can vary from year to year depending on several factors, but there is a general consensus among Brazilian antivenom producing Institutions that *Micrurus* venom availability is almost always lower than the desirable. In Brazil, coral antivenom is produced by Ezequiel Dias Foundation (FUNED), in Minas Gerais state, and by Butantan Institute, in São Paulo state. It is produced using venoms from *M. corallinus* and *M. frontalis* species as immunogens. These venoms are mostly composed of three-finger toxins (3FTx) and phospholipase A2 (PLA2), which are considered the major responsible for the envenoming symptoms caused by coral snakes. As these toxins are capable of inducing polyclonal antibodies (13), our group has identified neutralizing B-cell linear derived from them. These epitopes selection was made using the SPOT technique. Overlapping pentadecapeptides covering *M. corallinus* toxin's sequences were synthesized in a cellulose membrane and probed with anti-*M. corallinus* venom rabbit serum, FUNED and Butantan's coral antivenoms. The most reactive peptide sequences were selected, refined by immunoinformatics using the EPITOPIA epitope prediction tool (26) and used for rabbit immunization. This approach showed that synthetic peptides mimicking toxin's epitopes can improve the generation of antivenoms against coral snakes (14).

Building upon this previous approach, the present work proposes a new immunization protocol for Brazilian coral antivenom production, with a substantial reduction in the use of crude venoms, in a rabbit model. Three priming doses of *M. frontalis* venom and no *M. corallinus* venom at all would be necessary for this protocol, that was capable of eliciting neutralizing antibodies against these venoms. When compared to our previous work, which used only synthetic peptides as immunogens (14), the present protocol, using venom priming, showed an upgrade, achieving better antibody titers, neutralization potential and cross-reactivity. The produced anti-Ven_fro_/Pep_cor_ sera could fully protect animals challenged with a lethal dose of *M. corallinus* venom from death, whereas the previously produced anti-peptide sera promoted half of this protection.

Another novelty of the present work was that neutralization properties of the experimental antivenom was also tested with *M. frontalis* venom. Anti-Ven_fro_/Pep_cor_ antibodies neutralized PLA2 activity of *M. frontalis* venom and promoted 50% lethality protection of animals challenged with this venom. The fact that *M. frontalis* venom was less neutralized by our experimental antivenom than *M. corallinus* venom is noteworthy, considering that ELISA reactivity suggests that the elicited antibodies recognize *M. frontalis* venom better than *M. corallinus* venom. One possibility to explain the lack of *M. frontalis* venom neutralization by Anti-Ven_fro_/Pep_cor_ antibodies is the fact that, although we have used the amount related to 1.5 LD_50_ for both venoms in the performed challenge, the absolute venom amounts used are not the same. We considered in the assay the intra peritoneal LD_50_ values published by Tanaka et al. (18), being the value for *M. corallinus* venom 7 and 22 μg for *M. frontalis* venom, per 20 g of mice. Thus, in the assay, we injected a total of 10.5 μg of *M. corallinus* venom and 33 μg of *M. frontalis* venom, i.e., the absolute amount of *M. frontalis* venom injected in the animals was more than the triple of the amount used for *M. corallinus* venom. LD_50_ values may vary between venom batches. In the literature and in previous studies from our group, we can find intraperitoneal LD_50_ values for *M. frontalis* venom in mice ranging from 4 to 29 μg and from 5 to 27 μg for *M. corallinus* venom (27). As coral venoms are obtained in minor amounts, we chose to use values from the literature rather than establish the LD50 for our venoms experimentally. We chose Tanaka's work as it deals with both *M. frontalis* and *M. corallinus* venoms, allowing to use the same reference for both venoms. However, the real LD_50_ for our used venoms might have been different. Another possibility is that the immunological response of the host was started with *M. frontalis* venom as antigen, but affinity maturation may have occurred toward *M. corallinus* peptides. Therefore, in the final sera, anti-peptide antibodies may have prevailed rather than anti- *M. frontalis* venom.

Despite this incomplete neutralization toward *M. frontalis* venom, the present proposal was efficient as a proof-of-concept and there are still many approaches that can be readily used to improve it. The peptides used for substituting *M. corallinus* venom derived from sequences of only 3FTx and PLA2 toxins. These are indeed the most abundant components of *Micrurus* venoms (13), but there are other venom components that, despite being present in smaller amounts, can have an important role in envenoming, such as l-amino acid oxidases, metalloproteinases, c-type lectins, etc. (28). As demonstrated by Western Blot results (Figure 5), there are indeed venom protein bands with weaker binding by anti-Ven_fro_/Pep_cor_ sera. Peptides mimicking epitopes from these other toxin families can be incorporated into the immunization protocol to broaden antibodies reactivity and increase neutralizing potential. Also, as an improvement prospect, the immunization scheme used here can be altered, to represent *M. frontalis* venom in more doses along the program.

The proposed alternative protocol for coral antivenom is still a preliminary work that needs to be further validated, but extrapolating the obtained results to real situations reveals that the presented results are promising. Considering the body mass proportion between a 20 g-mouse and a 70 kg-human, our neutralization assay simulates the inoculation of 36.75 mg of *M. corallinus* venom or 115.5 mg of *M. frontalis* venom to a victim, which exceeds by far the amount of venom a *Micrurus* snake can inject (8). For antivenom quality control, Brazilian guidelines states that coral antivenom may have a maximum protein amount of 150 mg per mL that should be able to neutralize 1.5 mg of *M. frontalis* venom. Therefore, to be considered for clinical use, anti-Ven_fro_/Pep_cor_ sera should be able to neutralize 13.5 μg of *M. frontalis* venom in a neutralization assay [considering that 100 μL of rabbit serum contains 1.325 mg of IgG (29)]. As the experimental antivenom (anti-Ven_fro_/Pep_cor_ sera) was able to neutralize 50% of the lethality caused by 33 μg of *M. frontalis* venom, more than twice the required venom amount in antivenom quality control, we can consider that this protocol may be an interesting approach to coral antivenom production. We must consider carefully the situation illustrated above, since several factors such as different venom susceptibility between mice and humans and the nature of experimental and currently produced antivenom (crude rabbit serum and purified horse Fab'2, respectively) may separate the theoretical observation from reality.

*M. frontalis* and *M. corallinus* are the most common coral snakes found in Brazil, but severe accidents with different *Micrurus* species have been reported in Brazilian territory (30, 31). *In vitro* experiments demonstrated that the currently produced coral antivenom does not recognize adequately some venom components of other *Micrurus* species and may also poorly neutralize them (12, 32–34). Tanaka et al. showed, in 2010, (18) that coral antivenom produced by Butantan was not completely effective in neutralizing enzymatic activities from Brazilian *Micrurus* venoms. Remarkably, Butantan's coral antivenom was unable to completely neutralize PLA2 activity from *M. frontalis* venom, which is present in the immunization mixture used to produce the antivenom, whereas anti-Ven_fro_/Pep_cor_ sera developed in the present work was able to do so. It is also noteworthy in this previous work that Butantan's antivenom was not completely effective in neutralizing lethality from several *Micrurus* species (*M. altirostris. M. lemniscatus, M. spixii)*, including *M. corallinus*, which is also included in the immunization pool. In another work from this same group (34), the immunogenicity of several *Micrurus* venoms was assessed, aiming at finding experimental basis for broadening coral antivenom reactivity. Monovalent sera toward *M. altirostris, M. corallinus, M. frontalis, M. lemniscatus, M. spixii* and a polyvalent serum were produced in horse. Neutralization assays performed with these produced sera showed that none of the tested immunization approaches were completely efficient, indicating that finding an ideal immunogen for coral antivenom production in Brazil is yet an unsolved issue. Confirming the low cross-neutralization of Brazilian coral antivenom, a clinical case report tells that the available therapeutic antivenom was not completely efficient in reversing the symptoms of patients bitten by *Micrurus* species other than the ones used in the immunization pool, even when administered early (31).

With this in view, achieving better cross-neutralization becomes an important goal for improving treatment of coral snake envenomed victims (7). Anti-Ven_fro_/Pep_cor_ sera showed good cross-recognition of different relevant Brazilian *Micrurus* venoms in ELISA, although this does not necessarily mean cross-neutralization of toxic activities (34). But still, this is an important feature of anti-Ven_fro_/Pep_cor_ sera that must be further explored and improved if necessary.

The main toxin families are conserved among Brazilian *Micrurus* venoms but there is a large variation in the individual molecules itself, probably reflecting different evolutionary adaptations to habitats, preys and predators (18). Also, less abundant venom components can differ substantially between *Micrurus* species (28). If an efficient coral antivenom cross-neutralization with different *Micrurus* species is pursued, a wider variety of molecules should be represented in the immunization mixture used in the antivenom production process.

The use of synthetic epitopes to substitute venoms in antivenom production is not novel. Our group has been working on this theme for several years, with promising results (14, 35–38). An approach similar to the present work was proposed and validated to produce brown recluse spider antivenom in horses, combining crude venom and a recombinant synthetic antigen, containing epitopes previously mapped in relevant *Loxosceles* spp. toxins. The combined protocol achieved neutralization parameters comparable to that obtained with venom exclusively and better than using the synthetic antigen alone. This combined protocol reduced by 67% the need for using crude venom for brown recluse spider antivenom production (39).

Venomics and antivenomics studies are increasing venom composition knowledge (40–42). Immunochemical studies that select potential epitopes to be represented as synthetic peptides or recombinant proteins may also circumvent the lack of immunogenicity observed for some toxins, including the most prevalent 3FTX and PLA2 toxins (43). This increasing knowledge plays an important role in achieving better coverage of coral antivenom, leading to a possible pan-specific antidote toward *Micrurus* venoms (44).

## Concluding Remarks

Our results show that it is possible to produce cross-reactive, neutralizing coral antivenom substituting *M. corallinus* venom by synthetic peptides derived from relevant toxin sequences. This implicates in a reduced dependency on venom availability for the production of antivenom and the possibility of manipulating cross-reactivity, by adding other desired toxin-epitopes. This preliminary step can lead to enhanced production of better antivenoms, addressing the important issue of antivenom shortage and may lead toward the development of a pan-American antivenom.

In addition of the benefits for antivenom fabrication, decreasing the usage of venom for coral antivenom production would allow more venom to be assigned for studies aiming at describing *Micrurus* envenoming pathophysiology better and should also foment the disclosure of the biotechnological potential of *Micrurus* venoms.

## Data Availability Statement

The datasets generated for this study are available on request to the corresponding author.

## Ethics Statement

The animal study was reviewed and approved by Ethics Committee in Animal Experimentation from the Federal University of Minas Gerais.

## Author Contributions

CC-O, CF, PH, and CG-D: conception and design of study. KC, LL, DO, and RM: acquisition of data. KC, LL, and CG-D: analysis and interpretation of data. CF and CC-O: contribution of reagents, materials, and analysis tools. CG-D and AP: drafting the article. KC, LL, AP, PH, CC-O, and CG-D: revised critically the final version. All authors approved the final version of the submitted manuscript.

### Conflict of Interest

The authors declare that the research was conducted in the absence of any commercial or financial relationships that could be construed as a potential conflict of interest.
